# Development of a Diagnostic Marker for *Phlebotomus papatasi* to Initiate a Potential Vector Surveillance Program in North America

**DOI:** 10.3390/insects9040162

**Published:** 2018-11-12

**Authors:** Austin Merchant, Tian Yu, Jizhe Shi, Xuguo Zhou

**Affiliations:** Department of Entomology, University of Kentucky, Lexington, KY 40546, USA; ajme232@g.uky.edu (A.M.); tyty@bu.edu (T.Y.); jizhe.shi@uky.edu (J.S.)

**Keywords:** *Phlebotomus papatasi*, sand fly-borne diseases, PCR-based diagnostic assay, vector surveillance, sensitivity and specificity

## Abstract

*Phlebotomus papatasi*, an Old World sand fly species, is primarily responsible for the transmission of leishmaniasis, a highly infectious and potentially lethal disease. International travel, especially military rotations, between domestic locations and *P. papatasi*-prevalent regions in the Middle East poses an imminent threat to the public health of US citizens. Because of its small size and cryptic morphology, identification of *P. papatasi* is challenging and labor-intensive. Here, we developed a ribosomal DNA-polymerase chain reaction (PCR)-based diagnostic assay that is capable of detecting *P. papatasi* genomic DNA from mixed samples containing multiple sand flies native to the Americas. Serial dilution of *P. papatasi* samples demonstrated that this diagnostic assay could detect one *P. papatasi* from up to 255 non-target sand flies. Due to its simplicity, sensitivity and specificity, this rapid identification tool is suited for a long-term surveillance program to screen for the presence of *P. papatasi* in the continental United States and to reveal geographical regions potentially vulnerable to sand fly-borne diseases.

## 1. Introduction

Leishmaniasis, a vector-borne disease caused by protozoans, is an often-neglected illness endemic to a total of 98 primarily tropical and subtropical countries. It is estimated that around 2 million new cases of leishmaniasis occur each year, the majority of which occur in South America, East Africa and the Middle East [1]. Of the primary forms of leishmaniasis, the most important are its cutaneous and visceral forms. Cutaneous leishmaniasis (CL) is the most common form of the disease and can cause severe skin lesions and permanent scarring. Visceral leishmaniasis (VL) is responsible for the majority of leishmaniasis-linked deaths and can damage the immune system and lead to deadly complications if untreated [2]. Leishmaniasis is caused by trypanosomes of the genus *Leishmania* and is transferred to humans through the bite of an infected female sand fly. Sand flies are a group of morphologically challenging-to-distinguish species that includes the major vectors of leishmaniasis. Specifically, 98 sand fly species are known or suspected to act as vectors of leishmaniasis, all belonging to the genera *Phlebotomus* and *Lutzomyia* (found in the Old World and New World respectively) [3]. They are small, rarely exceeding a length of 3.5 mm, and noiseless, rendering their attacks on hosts largely undetectable [4]. In addition, symptoms of leishmaniasis normally develop 2 to 8 months after being bitten, obscuring the link between the bite of a sand fly and the onset of disease. Considering the threat posed by leishmaniasis, it is important to establish vector surveillance programs for Phlebotomine sand flies in regions where they are suspected to exist or become established.

Although leishmaniasis is generally considered a tropical disease, a steady range expansion has been observed in both the parasite itself and the sand flies that vector it. Of the New World sand flies, *Lutzomyia shannoni* has the largest geographic distribution, ranging from Argentina to the southeastern United States [5]. Laboratory studies have shown that *L. shannoni* is capable of transmitting *Leishmania panamaensis* [6] and *Leishmania mexicana* [7], both of which cause CL. A 2010 survey on the distribution of sand flies in Kentucky recorded the presence of *L. shannoni* in the area, indicating a northward expansion of its habitat towards the central US [8]. Similarly, the range of *Leishmania* has also expanded throughout regions of Texas, where it is now considered endemic [9,10]. Cases of leishmaniasis have also been reported in the states of Oklahoma [11] and North Dakota [12]. This range expansion coincides with the discovery of Old World sand flies and *Leishmania* species in previously non-endemic regions of Europe [13,14,15]. An increase in cases of canine leishmaniasis has also been observed in Europe, which is of concern in part because dogs act as reservoirs from which the disease can spread to humans [16]. Furthermore, simulation studies focused on the future distribution of sand flies in regions such as North America [17], Central America [18], Europe [19] and southwestern Asia [20] suggest that an increase in temperature and humidity, due to global warming, can allow flies to survive in previously uninhabitable areas. While climate change is generally viewed as a major factor in current and future sand fly range expansion, it should be noted that other factors—such as increased cross-border travel and changes in reservoir populations—are more likely to have immediate impacts.

In the United States, military involvement in the Middle East has raised public health concerns involving the introduction of sand fly-vectored diseases. This region is home to *Phlebotomus papatasi*, the principal vector for cutaneous leishmaniasis in the Old World. *Phlebotomus papatasi* transmits *Leishmania major*, which requires both a sand fly and a mammalian host to complete its development. *Leishmania major* is ingested by *P. papatasi* in its amastigote form, where it replicates and is later injected into a mammalian host as a promastigote [21]. *Phlebotomus papatasi* comprises either a majority or a significant portion of sand fly trap captures in the Middle East and Egypt [22,23,24,25,26], including Iran where CL is especially prevalent [27,28,29]. Currently, *P. papatasi* has not yet been found in the New World. However, troop rotations between endemic regions and domestic military bases might introduce leishmaniasis to US soil [30]. From 2001 to 2006, around 1300 incidences of leishmaniasis were diagnosed in United States military personnel who were returning from Afghanistan and Iraq [31]. Infected exotic sand flies, such as *P. papatasi*, may be carried back to the continental US by returning patients, military equipment and supplies. Exotic species may further transmit the disease to local mammalian reservoirs, which could then spread *Leishmania* parasites to local sand fly populations such as *L. shannoni*. However, it is unknown whether New World sand fly species are competent vectors of *Le. major*. At least two native sand fly species, *L. shannoni* and *L. vexator*, were previously captured and identified at Fort Campbell, Kentucky [32]. Transport of infected sand flies from regions where leishmaniasis is endemic has the potential to expose portions of the US population to the threat of sand fly-vectored diseases.

Given the potential threat of introducing exotic sand fly species to the United States, in the short-term through global trade and international travel and in the long-term through global warming, it is important to establish a surveillance program to monitor the invasion of these disease vectors. Current taxonomic keys are based extensively on subtle morphological traits such as genitalia and the cibarium in the head, which require time and expertise to identify [5]. The overall goal of this study was to develop a fast, easy and cost-effective diagnostic assay for *P. papatasi* detection. To achieve this, we designed and tested a PCR-based diagnostic assay utilizing the *P. papatasi* salivary apyrase gene as a molecular marker and then examined its sensitivity in vivo and specificity in silico.

## 2. Materials and Methods

### 2.1. Sand Fly Collection and Storage

*Lutzomyia shannoni* and *Lutzomyia vexator* specimens were collected from field sites during 2008 at the Fort Campbell Army Installation near Clarksville, TN and the University of Kentucky’s Western Research and Education Center in Princeton, KY using standard Center for Disease Control (CDC) light traps (Model 512, John W. Hock, Gainesville, FL, USA). *Phlebotomus papatasi* specimens originating from Israel, Jordan, North Sinai and Turkey were obtained from lab colonies maintained by the Walter Reed Army Institute of Research in Maryland. Specimens of *Lutzomyia longipalpis* originating from Brazil were also obtained from a lab colony maintained by Kansas State University. *Lutzomyia longipalpis* was chosen as a third representative species that is native to the Americas, although its native range is confined to Central and South America.

Sand flies were stored in 95% ethanol upon removal from −20 °C storage. Individual specimens were temporarily removed from ethanol for dissection of the heads and last 2–3 abdominal segments. Dissected body parts were placed in separate wells of 0.25 mL PCR strip tubes along with approximately 0.2 mL of a lactic acid-phenol based commercial clearing solution (Bioquip Inc. Rancho Dominguez, CA, USA) for subsequent taxonomic identification. The remainder of each specimen was individually stored in centrifuge tubes with 95% ethanol and labeled with specimen accession numbers. Specimen vouchers of field collected material were retained in the collection of the Public Health Entomology Laboratory at the University of Kentucky.

### 2.2. Taxonomic Identification

The head and last 2–3 abdominal segments of each specimen were cleared and processed using a modification of the methods presented in Reference [33] with commercial clearing solution used as a substitute for boiling sodium hydroxide. The fly fragments were temporarily mounted on glass microscope slides for viewing at 20× magnification under a compound light microscope and then identified to species using a morphological key [34].

### 2.3. Genomic DNA Extraction and Sample Preparation

Remaining portions of specimens were individually dried in a rotary evaporator to remove ethanol. A single 2.5 mm glass bead was added to each tube along with 75 μL of PCR nanopure water. Tubes were placed in a Mini beadbeater (BioSpec Products Inc., Bartlesville, OK, USA) for 1.5 min of grinding. DNA slurries were mixed with 180 μL ATL lysis buffer (Qiagen Inc., Hilden, Germany) and 20 μL Proteinase K (Qiagen Inc.) and incubated overnight on a dry heating block at 56 °C. The standard DNAeasy Tissue Kit (Qiagen Inc.) extraction protocol was followed from this point on, ending with two final elutions in 100 μL of buffer AE. The DNA concentration of each sample was determined using a NanoDrop ND-1000 Spectrophotometer (Thermo Fisher Scientific, Waltham, MA, USA).

Individual sand fly DNA samples were sorted according to species identification. Artificially mixed samples of *L. shannoni*, *L. vexator* and *L. longipalpis* were made by combining individual samples of the same concentration from the three species with a 1:1:1 ratio. Sand fly DNA mixtures were prepared by adding one *P. papatasi* specimen to each of these mixed samples. Individual *P. papatasi* samples from Israel, Jordan, North Sinai and Turkey were subjected to a serial dilution with ddH_2_0 or sand fly DNA mixtures described above. 

### 2.4. Primer Design

The complete CDS of the mammalian-like lipase (accession number: AY179968) and salivary apyrase (accession number: AF261768) *Phlebotomus papatasi* mRNA sequences were obtained from NCBI. Both were subjected to Blastn analysis and searched against all insect nucleotide entries. Of the top 100 Blastn results, sequences belonging to the *Phlebotomine* sand flies, including the query sequence, were retained for multiple sequence alignment with MUltiple Sequence Comparison by Log-Expectation (MUSCLE) [35]. Three primer sets were designed for each mRNA within the regions with the lowest homogeneity. Table 1 shows the sequence for the primer sets along with the expected length of amplified products.

### 2.5. PCR Amplification

Sand fly DNA samples were amplified using the designed primer sets and iQ™ SYBR^®^ Green Supermix. Each reaction contained 10.0 μL of enzyme supermix, 1.0 μL of template DNA, 1.0 μL of forward primer and reverse primer each and 7.0 μL of ddH_2_0. PCR was performed in accordance with the supermix manufacturer’s protocol. PCR was carried out in a thermal cycler with the following cycling conditions: initial denaturing at 94 °C for 3 min; 40 cycles of 15 s denaturing at 94 °C, 30 s annealing at 55 °C and 30 s extension at 72 °C; and a final extension at 72 °C for 5 min. 

PCR products were validated using gel electrophoresis. Amplified products were visualized on 1.5% agarose gels stained with Gelred (Biotium Inc., Fremont, CA, USA, Cat. # 41002) in 0.5× TBE buffer, which was run at 80V for 30 min. A 1000 plus base pair DNA ladder (1KB plus Gene Ruler™, Fermentas Inc., Burlington, MA, USA, Cat. # SM1333) was used as the molecular size marker.

### 2.6. In Silico Analysis of the Specificity of the Selected Diagnostic Marker 

After being selected as the diagnostic marker, the specificity of *P. papatasi* salivary apyrase (APY) mRNA primer set 2 was analyzed bioinformatically. First, a nucleotide search was conducted on NCBI with the items “salivary” and “apyrase” and the results were restricted to Psychodidae. All returned sequences were aligned using MUSCLE [35] and edited by Mega7 [36]. Another MUSCLE alignment was conducted between the APY primer set 2 product and aligned sequences from the previous step using EMBL-EBI (European Bioinformatics Institute), to generate a percent identity matrix. Pair-wise comparison between the APY primer set 2 product and returned sand fly APY sequences was carried out following the resultant matrix. Second, the APY primer set 2 product was subjected to Blastn search against nucleotide entries in all organisms. Program selection was optimized to “Somewhat similar sequences (blastn).” Returned sequences were compared by query coverage and percent identity. Information regarding the sources of the returned sequences was summarized in Tables S1 and S2.

## 3. Results

**Figure 1:**
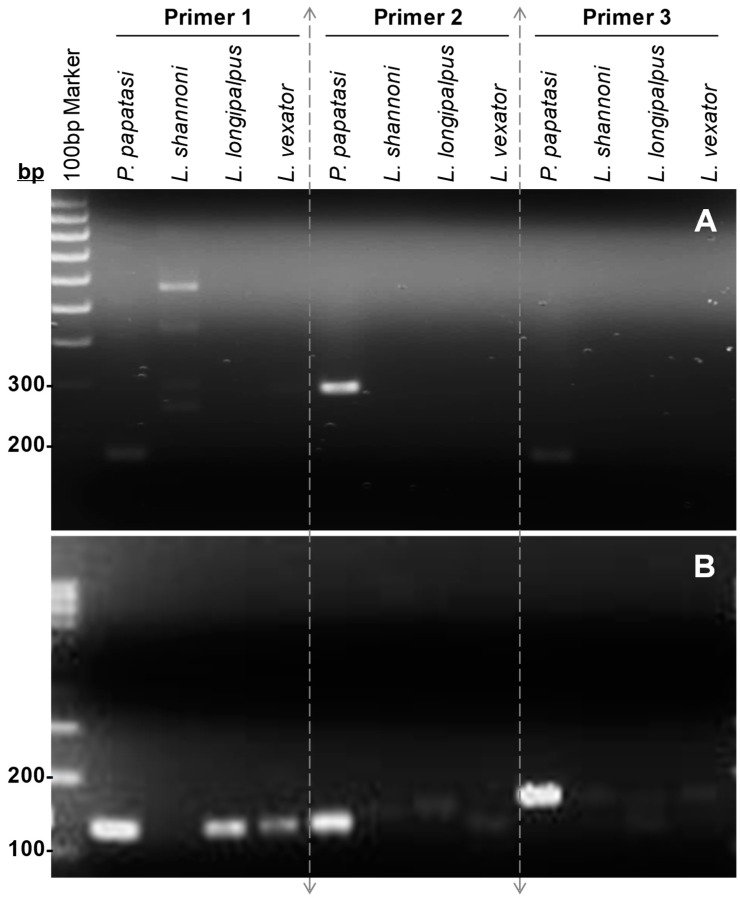
Selection of the diagnostic primer set for *Phlebotomus papatasi*. To search for *P. papatasi*-specific markers, two nucleus genes, salivary apyrase (accession number: AF261768); (**A**) and mammalian-like lipase (accession number: AY179968); (**B**) were used in this study. Three primer sets were designed for each mRNA within the regions with the lowest homogeneity. Primer sequences and projected amplicon sizes were listed in Table 1. Some bands of very low intensity were visible after running the gel but are not visible in photos. Notably, bands pertaining to all three non-target species appear when LIP primer set 3 is used.

In order to differentiate the exotic sand fly species *P. papatasi* from native *L. shannoni* and *L. vexator*, our candidate primer sets should be able to only amplify PCR products from *P. papatasi* genomic DNA. Figure 1 displays the band patterns for amplifications using different sand fly DNA and different primer sets. For reactions using the three mammalian-like lipase (LIP) primer sets, bands corresponding to *P. papatasi* can be visualized at approximately 130, 130 and 180 bp respectively. However, bands corresponding to non-target species were also observed in reactions using LIP primer sets 1 and 3. Reactions with salivary apyrase (APY) primer sets yielded bands at approximately 170, 230 and 170 bp for *P. papatasi*. Among them, amplified products of APY primer set 2 exhibited the most intense band for *P. papatasi* without additional bands for other species, such as the band for *L. shannoni* found in primer set 1. Therefore, this primer set was selected as the diagnostic marker for the subsequent sensitivity testing. 

**Figure 2:**
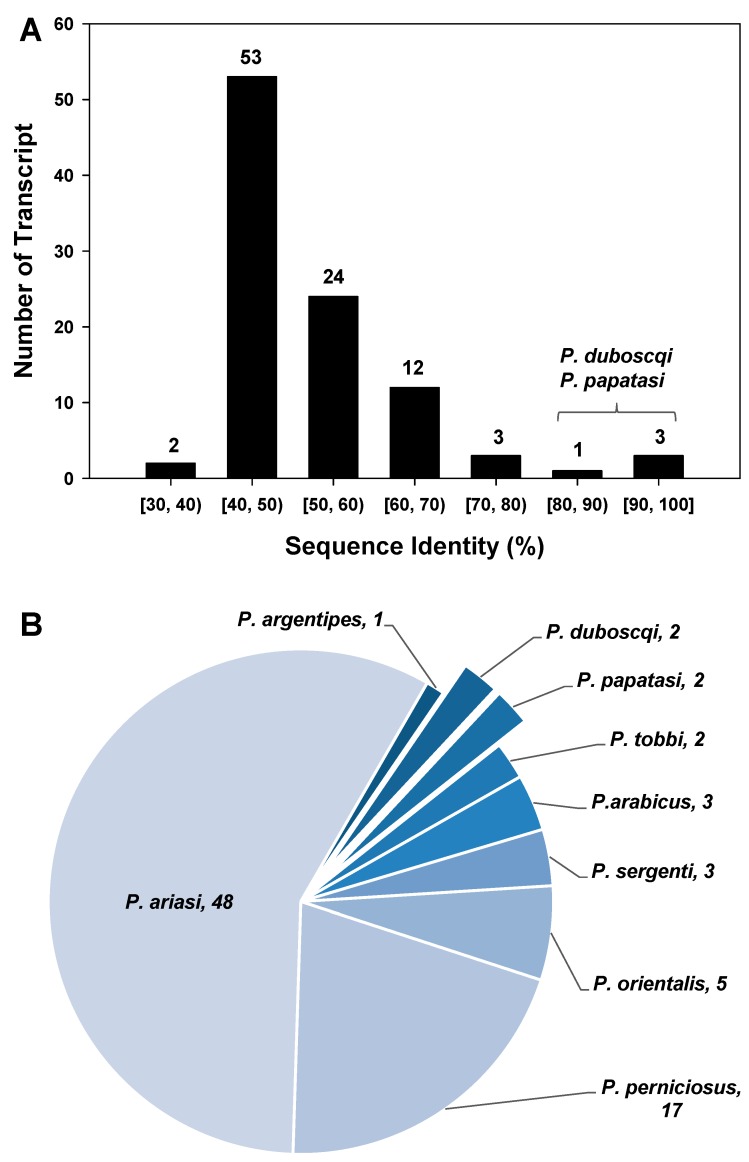
Specificity of *P. papatasi* salivary apyrase primer set 2. MUltiple Sequence Comparison by Log-Expectation (MUSCLE) was used to align and compare the sequence identity of the PCR product generated by the primer set 2 of *P. papatasi* salivary apyrase with 98 apyrase transcripts in other sand flies. (**A**) The distribution of the number of apyrase transcripts across different range of percent identity; (**B**) The number of apyrase transcripts from different species in *Phlebotomus*.

When APY primer set 2 was compared with all sand fly APY transcripts, 135 results were returned. After MUSCLE alignment and editing, 98 sequences remained. Most of the sequences ranged from 40% to 70% percent identity with the primer set 2 product, as shown in Figure 2. 53 sequences’ identities were between 40% and 50%, 24 were between 50% and 60% and 12 were between 60% and 70%. There were only 4 sequences with identities higher than 80%, all of them belonging to either *P. papatasi* or *P. duboscqi*. Information on the identities of the apyrase transcripts with the greatest sequence identity can be found in Table S1.

The Blastn results with the highest sequence identity are shown in Table S2. 33 results were returned from the Blastn search across 4 kingdoms: Animalia, Plantae, Fungi and Bacteria. 23 of the sequences were from animals, of which 12 sequences belonged to insects and half of the insects’ sequences belonged to sand flies. Among all returned results, only 4 of them had a more than 50% query coverage: 2 from *P. papatasi* (100%) and 2 from *P. duboscqi* (99%) with over 87% percent identity, which indicated a significant match with the APY primer set 2 product.

**Figure 3:**
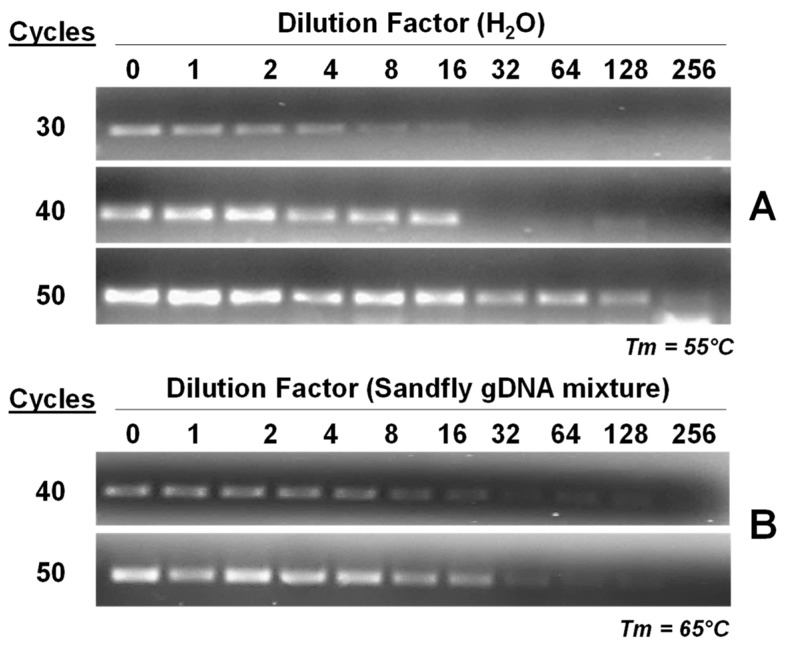
Sensitivity of PCR-based diagnostic assay. To examine the sensitivity of this diagnostic assay, *P. papatasi* samples were diluted into ddH_2_0 (**A**) or sand-fly DNA mixtures (**B**). In addition, the number of PCR cycles also contributed to the sensitivity of this rapid detection method. Dilution factor based on serial dilution ranged from 0- to 256-fold.

To test the sensitivity of the primer set, we investigated the effect of sample dilution on the amplification of PCR products. Figure 3 illustrates the visibility of PCR products amplified from *P. papatasi* DNA samples diluted with either ddH_2_0 or artificial DNA mixes of the three non-target native species (*L. shannoni*, *L. vexator* and *L. longipalpis*).

**Figure 4:**
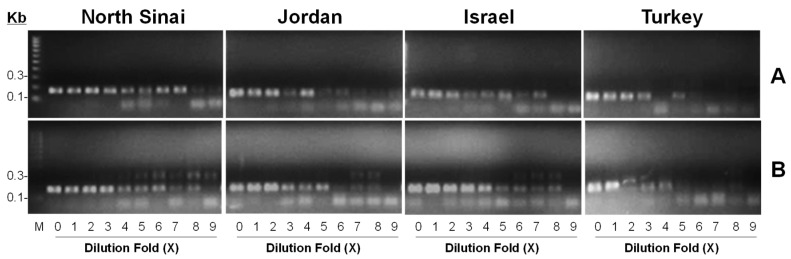
Validation of sensitivity. Field-collected *P. papatasi* samples from Israel, Jordan, North Sinai and Turkey were, respectively, subjected to a serial dilution with ddH_2_0 (**A**) or artificially mixed sand fly DNA samples (**B**) including *L. shannoni*, *L. vexator* and *L. longipalpis*. Dilution factor based on serial dilution comprised 0 (0), 1 (1), 2 (2), 3 (4), 4 (8), 5 (16), 6 (32), 7 (64), 8 (128) and 9 (256).

After 50 cycles, in both dilution strategies, bands of amplified product were visible up to the 256th-fold dilution level. Figure 4 illustrates the visibility of PCR products amplified from diluted *P. papatasi* DNA samples originating from Israel, Jordan, North Sinai and Turkey via APY primer set 2. Bands were visible at the 256th-fold dilution level in all but the Turkey sample, where bands were visible up to the 16th-fold dilution.

## 4. Discussion

Previously, members of our lab developed and tested a PCR-restriction fragment length polymorphism (RFLP)-based assay for the differentiation of sand fly species using the mitochondrial cytochrome oxidase 1 (CO1) gene [37]. Here, we demonstrate the use of a similar PCR-based assay as an effective form of surveillance in the detection of non-endemic *P. papatasi* sand flies. PCR-RFLP has been used extensively in regions where leishmaniasis is endemic as a means of identification for both sand fly [38,39,40,41,42] and *Leishmania* [27,28,29] species. In addition to PCR-RFLP, DNA barcoding [43,44,45,46,47] and matrix-assisted laser desorption/ionization time of flight mass spectrometry [48,49,50] have also been used to differentiate sand fly species. In comparison to other methods of identification, the PCR basis of our assay removes the need for DNA sequencing and requires only rudimentary equipment to perform. In addition, it is tuned specifically towards detection of *P. papatasi* sand flies, which allows the assay to produce completely unambiguous results. Finally, our assay can detect *P. paptasi* DNA even when it is homogenized with other insect samples, making it ideal as a means of monitoring *P. papatasi* presence using light trap captures.

Previous studies using *P. papatasi* revealed that mammalian-like lipase (LIP) is a major protein involved in the secretions of female reproductive accessory glands [51]. Considering that the anatomy of internal reproductive organs is used for sand fly species identification, differences in morphology may reflect on the mRNA sequence of the LIP protein. Research on salivary apyrase (APY) in *P. papatasi* also indicates that this protein family is highly diverse in hematophagous arthropods [52]. For both proteins, their corresponding mRNAs can be potentially utilized for species identification. Blast searches against insect nucleotide entries generally resulted in low homogeneity with other species, with the only exception being that *P. papatasi* and *P. duboscqi* from Mali share 90% identity with the APY mRNA. This similarity has been verified previously [53]. *Phlebotomus duboscqi*’s native range overlaps with that of *P. papatasi* and it is also capable of carrying *Leishmania* [3,54]. Thus, although it is likely that our surveillance system yields a positive result with *P. duboscqi* DNA, this exception is acceptable due to the risk posed by this species.

Multiple sequence alignment for mammalian-like lipase (LIP) and salivary apyrase (APY) of *P. papatasi* and related sand fly species revealed conserved domains among closely related sand fly species. As denoted in Figure 4, primer sets were preferably selected at disparate regions in the alignment. If the conserved region was overwhelmingly long as in the case of salivary apyrase, high-scoring segment pair (HSP) regions between *P. papatasi* and non-target species would be used as the secondary selection criteria. This design strategy is intended to improve the probability of obtaining species-specific primer sets at the bioinformatics level.

Candidate primer sets were evaluated on DNA samples of four sand fly species to ensure that a sufficient level of specificity was achieved to distinguish *P. papatasi* from non-target species. APY primer set 2 was able to amplify DNA samples of *P. papatasi* but no other species, indicating a strong and specific binding of the primer set to *P. papatasi* DNA. Other primer sets yielded bands exhibiting various degrees of anomaly including weak intensity, the presence of multiple bands and band deformation. These anomalies were probably due to unspecific binding to DNA samples of non-target species. The length of amplified products was generally in accordance with the expected values predicted from mRNA sequences, indicating the absence of introns and splicing variants that could affect primer positions.

Subsequently, we investigated the sensitivity of APY primer set 2 by diluting *P. papatasi* samples with ddH_2_0 and DNA mixes of non-target sand flies. *Phlebotomus papatasi* is a sand fly species which poses an invasion threat to the US. At the initial stage of a potential invasion, the number of *P. papatasi* that are present in CDC traps would be extremely limited, which poses a great challenge to its detection from a pool of insect DNA mixtures. Nevertheless, APY primer set 2 was able to detect and amplify *P. papatasi* originating from most regions up to the ninth dilution level, indicating an equivalent identification power of detecting one *P. papatasi* from 255 native sand fly individuals. Due to possible contamination of the Turkey DNA sample, bands above the fifth dilution level were not readily visible, which is equivalent to detecting one *P. papatasi* from 15 native sand fly individuals. Compared to the clear gel background of the ddH_2_0 dilution group, some barely visible bands at 300 bp were present in the DNA mix dilution group. While the intensity of the target band at around 140bp was not greatly affected, the presence of irreverent bands illustrates the influence of non-target DNA on *P. papatasi* detection.

## 5. Conclusions

In conclusion, our PCR-based assay can successfully detect the presence of *P. papatasi* genomic DNA from DNA mixtures consisting of native sand fly species. Our next goal is to apply our assay to captures from standard CDC light traps acquired from Spindletop Farm, a research farm located near the University of Kentucky, and Fort Campbell, Kentucky as a means of field validation. If successful, development of a mobile field kit is also a possibility, to allow technicians to analyze trap captures on-site. Furthermore, it may also be possible to adapt our assay for use in detecting exotic sand fly species in other regions at risk of invasion, including Europe and the Middle East. The most effective time window for any surveillance strategy is prior to or during the initial stage of invasion when introduced populations of non-native species have yet to become established. Using readily available PCR technologies, our detection strategy achieves significant levels of sensitivity and specificity while theoretically reducing the time, labor, cost and expertise required by traditional surveillance strategies based on either morphological traits, molecular features, or both.

## Figures and Tables

**Table 1:** Sequences of primer sets tested for diagnostic use, with expected amplicon length.

Gene	Accession Number	Primer	Sequence (5′-3′)	Amplicion Length (bp)
*Mammalian-like lipse*	AY179968	LIP1F	CTGCGAGGCCAACGTGGACA	132
		LIP1R	GCGCAGAGGTCACAGAGGTCG	
		LIP2F	CCGGCCACTACGGTGTTGAGG	135
		LIP2R	CCAGTTCCGACGATCGATTT	
		LIP3F	ACGTCACACTCTTCCCCAAC	172
		LIP3R	TAGTCGCACTTGGCCTTCTT	
*Salivary apyrase*	AF261768	APY1F	ACAAAGGACGAGGAGCTGAA	178
		APY1R	GTTGCCCATTCTGCCTTAAA	
		APY2F	TGGCACGAAGCTGTTAATTG	228
		APY1R	GTCA TCAC TATC GGGG AGGA	
		APY3F	ACCA ATGC AGAC CTCA TTCC	174
		APY3R	TTTG ATCC AGAG GGAG TTGC	

## Supplementary Materials

The following are available online at http://www.mdpi.com/2075-4450/9/4/162/s1, Table S1: Sequence comparison between *P. papatasi* salivary apyrase primer set 2 product and apyrase transcripts in other sand flies, Table S2: Sequence comparison between *P. papatasi* salivary apyrase primer set 2 product and top blastn hits.
