# Supplementary material for: Development of a Diagnostic Marker for Phlebotomus papatasi to Initiate a Potential Vector Surveillance Program in North America
Source: Insects. 2018 Nov 12;9(4):162. doi: 10.3390/insects9040162 (PMC6315872; doi:10.3390/insects9040162)
Supplement: Supplementary file 1 [file insects-09-00162-s001.pdf]

**Table S1**. Sequence comparison between *P. papatasi* salivary apyrase primer set 2 product and apyrase transcripts in other sandflies.

| Genus | Species * | Accession No. | Identity [a] (%) | Coverage [b] (%) |
|---|---|---|---|---|
| *Phlebotomus* | *papatasi* | AF261768.1 | 100.0 | 100.0 |
| *Phlebotomus* | *duboscqi* | DQ834335.1 | 91.2 | 100.0 |
| *Phlebotomus* | *sergenti* | HM560862.1 | 78.2 | 100.0 |
| *Nyssomyia* | *intermedia* | KA660068.1 | 64.5 | 100.0 |
| *Phlebotomus* | *orientalis* | KC170936.1 | 63.2 | 100.0 |
| *Phlebotomus* | *argentipes* | DQ136150.1 | 62.0 | 96.1 |
| *Phlebotomus* | *perniciosus* | DQ192491.1 | 61.8 | 100.0 |
| *Phlebotomus* | *ariasi* | AY845193.1 | 61.4 | 100.0 |
| *Phlebotomus* | *tobbi* | HM135951.1 | 61.0 | 100.0 |
| *Phlebotomus* | *arabicus* | EZ000631.1 | 60.5 | 100.0 |
| *Lutzomyia* | *ayacuchensis* | AK416757.1 | 58.8 | 100.0 |
| *Lutzomyia* | *longipalpis* | AF131933.1 | 57.5 | 100.0 |

*: If sand fly species have multiple apyrases, the transcript with the highest sequence identity was included in this table. [a]: the percentage of query with a direct match in the alignment to the *P. papatasi* salivary apyrase primer set 2 product sequence. [b]: the percentage of query covered by alignment to the *P. papatasi* salivary apyrase primer set 2 product sequence.

**Table S2.** Sequence comparison between *P. papatasi* salivary apyrase primer set 2 product and top blastn hits.

| Kingdom | Order | Genus | Species | Accession # | Identity [a] (%) | Coverage [b] (%) |
|---|---|---|---|---|---|---|
| Animalia | Diptera | *Phlebotomus* | *papatasi* | AF261768.1 | 100.0 | 100.0 |
| Animalia | Diptera | *Phlebotomus* | *papatasi* | JQ988892.1 | 98.7 | 100.0 |
| Animalia | Diptera | *Phlebotomus* | *duboscqi* | DQ834335.1 | 91.6 | 99.0 |
| Animalia | Diptera | *Phlebotomus* | *duboscqi* | DQ834331.1 | 87.7 | 99.0 |
| Animalia | Diptera | *Bichromomyia* | *olmeca* | KX011391.1 | 73.5 | 43.0 |
| Animalia | Diptera | *Lutzomyia* | *longipalpis* | AF131933.1 | 75.8 | 28.0 |
| Animalia | Diptera | *Stomoxys* | *calcitrans* | XM_013248487.1 | 80.7 | 24.0 |
| Animalia | Strongylida | *Haemonchus* | *contortus* | LS997564.1 | 78.2 | 24.0 |
| Animalia | Chiroptera | *Hipposideros* | *armiger* | XM_019631939.1 | 85.0 | 17.0 |
| Animalia | Chiroptera | *Hipposideros* | *armiger* | XM_019631940.1 | 85.0 | 17.0 |
| Animalia | Chiroptera | *Hipposideros* | *armiger* | XM_019631941.1 | 85.0 | 17.0 |
| Animalia | Chiroptera | *Hipposideros* | *armiger* | XM_019631942.1 | 85.0 | 17.0 |
| Animalia | Tricladida | *Dugesia* | *japonica* | AB610879.1 | 85.0 | 17.0 |
| Fungi | Schizosaccharomycetales | *Schizosaccharomyces* | *octosporus* | XM_013164291.1 | 87.5 | 17.0 |
| Animalia | Apterygiformes | *Apteryx* | *australis* | LK066971.1 | 86.8 | 16.0 |
| Animalia | Apterygiformes | *Apteryx* | *australis* | LK235606.1 | 86.8 | 16.0 |
| Animalia | Diptera | *Lucilia* | *cuprina* | XM_023439892.1 | 84.2 | 16.0 |
| Plantae | Solanales | *Ipomoea* | *triloba* | CP025662.1 | 84.2 | 16.0 |
| Animalia | Cypriniformes | *Danio* | *rerio* | CU468826.10 | 86.1 | 15.0 |
|  | Bacillales | *Bacillus* | *pumilus* | CP027116.1 | 86.1 | 15.0 |
| Animalia | Coleoptera | *Aethina* | *tumida* | XM_020009867.1 | 93.8 | 14.0 |
| Animalia | Coleoptera | *Aethina* | *tumida* | XM_020009868.1 | 93.8 | 14.0 |
| Animalia | Coleoptera | *Aethina* | *tumida* | XM_020009869.1 | 93.8 | 14.0 |
| Animalia | Coleoptera | *Aethina* | *tumida* | XM_020009870.1 | 93.8 | 14.0 |
| Animalia | Artiodactyla | *Bos* | *mutus* | CP027089.1 | 87.9 | 14.0 |
| Animalia | Artiodactyla | *Ovis* | *canadensis* | CP011903.1 | 87.9 | 14.0 |
| Bacteria | Synechococcales | *Prochlorococcus* | *marinus* | CP000095.2 | 88.6 | 14.0 |
| Bacteria | Clostridiales | *Blautia* | *sp.* | CP030280.1 | 93.3 | 13.0 |
| Plantae | Lamiales | *Erythranthe* | *guttatus* | XM_012988858.1 | 90.3 | 13.0 |
| Plantae | Malvales | *Theobroma* | *cacao* | LT594797.1 | 90.3 | 13.0 |
| Plantae | Malvales | *Theobroma* | *cacao* | XM_007010613.2 | 90.3 | 13.0 |
| Plantae | Malvales | *Theobroma* | *cacao* | XM_018129620.1 | 90.3 | 13.0 |
| Plantae | Malvales | *Theobroma* | *cacao* | LT594792.1 | 92.9 | 12.0 |

Shaded region denotes transcript with > 50% coverage. [a]: the percentage of query with a direct match in the alignment to the *P. papatasi* salivary apyrase primer set 2 product sequence. [b]: the percentage of query covered by alignment to the *P. papatasi* salivary apyrase primer set 2 product sequence.
